# Does active case finding detect TB early in programme settings? A national-level study in India

**DOI:** 10.5588/pha.25.0044

**Published:** 2026-03-06

**Authors:** H.D. Shewade, S. Kiran Pradeep, P. Ravichandran, G. Kiruthika, A.N. Shah, B. Vadera, V. Roddawar, S.K. Mattoo, S. Iyer, D. Tumu, A. Chowdhury, S. Devika, J. Chadwick, R.R. Vaidya, P. Singh, S.K. Panda, M.A. Baig, K.V. Suma, M. Suleka, A.K. Digal, D. Banerjee, M.L. Prasanna, D.Y. Waghela, A. Krishnaraj, P. Kashyap, J.S. Parmar, C.K. Mishra, S. Das, A. Kumar, A. Kumar, S. Yadav, S. Chetry, A. Kumar, M. Pathak, S. Singh, S. Tabrez, P. Mehra, S. Ramesh, B. Bishnu, G. Mahesh, A. Rajesham, B.K. Mishra, U. Chandra Tripathi, K.U. Khayyam, K. Rade, R. Rao, M.V. Murhekar

**Affiliations:** 1 ICMR National Institute of Epidemiology (ICMR-NIE), Chennai, India;; 2 USAID India, New Delhi, India;; 3 John Snow India Private Limited, New Delhi, India;; 4 Central TB Division, Ministry of Health and Family Welfare, New Delhi, India;; 5 Office of the World Health Organization Representative to India, New Delhi, India;; 6 State TB Cell, Government of Gujarat, Ahmedabad, India;; 7 State TB Cell, Government of Uttarakhand, Dehradun, India;; 8 State TB Cell, Government of Telangana, Hyderabad, India;; 9 State TB Cell, Government of Bihar, Patna, India;; 10 National Institute of Tuberculosis and Respiratory Diseases, Government of India, New Delhi, India.

**Keywords:** tuberculosis, community-based systematic screening, early detection, marginalised populations, India

## Abstract

**SETTING:**

Since 2017, India’s TB programme is implementing active case finding (ACF) in high-risk populations in all districts. Symptom screening followed by confirmatory testing was the ACF algorithm.

**OBJECTIVE:**

To determine differences in pre-treatment delays and severe illness at diagnosis between ACF- and passive case finding (PCF)-detected adults with drug-sensitive pulmonary TB in high-risk populations.

**DESIGN:**

Cross-sectional analytical study from 28 randomly sampled districts across India (2023). Post-triaging, severe illness was defined as presence of very severe undernutrition, respiratory insufficiency, or poor performance status.

**RESULTS:**

Of 790 enrolled, 426 were ACF-detected and 364 PCF-detected. ACF-detected adults were significantly older (mean 47.1 year vs 43.9 year), lived farther from diagnosis facilities (median 8 km vs 6 km), had lower formal education exposure (52% vs 37% with no formal education), lower household income (₹20,000 vs ₹24,000 annual per capita), and experienced fewer health care provider visits (median 1 vs 2). Pre-treatment delay from symptom onset to treatment initiation (median 46 days in both groups) and burden of severe illness (39% vs 34%, *P* = 0.180) were similar.

**CONCLUSION:**

Though ACF linked the vulnerable to care and reduced health care provider visits, this did not translate into early detection. High burden of severe illness at diagnosis is a concern.

TB is the leading infectious disease killer globally, with an annual estimated 10.7 million new incident TB and 1.1 million TB-related deaths.^1^ National programmes have predominantly relied on passive case finding (PCF), which involves identifying people with TB (PwTB) at health care facilities among individuals who recognise their symptoms and proactively seek medical attention. Despite extensive efforts, 2.4 million PwTB are estimated to be undetected annually (gap between estimated and notified TB).^1–4^ The World Health Organization recommends active case finding (ACF) in addition to ongoing PCF.^2,5^ ACF involves systematic screening for active TB outside health care facilities in high-risk populations having at least 0.5% undetected TB prevalence.^5^ One of the key anticipated benefits of ACF is early detection.^6^ PwTB detected early may be initiated on treatment sooner, therefore potentially reducing the morbidity and mortality associated with delayed detection.^2,5^ A systematic review from 2013 suggested that screening found PwTB earlier and with less severe disease, but this might be due to more sensitive diagnostic methods used in research studies compared to routine programme settings using PCF.^6^ There is limited evidence from nationally representative studies conducted in routine health system settings where ACF is implemented by national TB programmes.

India bears the highest TB burden globally with 0.3 million missed PwTB (year 2023).^1,7,8^ In the past, the Global Fund–supported project *Axshya* implemented TB ACF in ≈285 districts across India (as of 2016–17). In these project settings, while ACF improved TB detection in short term, and reduced health system–level diagnosis delays and catastrophic costs due to TB diagnosis, it did not influence total pre-treatment delay and treatment outcomes.^9–13^ Following this, since 2017, India’s National TB Elimination Program (NTEP) has been implementing ACF in high-risk populations across all districts (see Supplementary Data Table S1 and Figure S1).^14,15^ COVID-19 represented a potential setback to India’s plan to achieve the Sustainable Development Goal TB targets.^8^ Intensive community engagement and ramped-up ACF was required to compensate for missed diagnoses during lockdown periods.^16^ Annual TB notifications reached pre-COVID-19 levels by 2022.^17,18^ Since the implementation of ACF in all districts by India’s NTEP, the benefits in terms of early detection have not been systematically assessed. To understand this and other aspects, India’s NTEP commissioned a national-level TB ACF evaluation (2022–24) to guide evidence-based strategic planning. Published findings suggest that ACF scale and quality are below the benchmarks with extent of use of rapid molecular diagnostic tests among ACF-detected presumptive TB being similar to PCF settings.^19,20^ Qualitative systematic enquiry recommended six tips to address the ‘know’ and ‘know-do’ gap.^21^ In this manuscript, we present our findings related to early TB detection.

In this nationally representative study from 2023, we aimed to determine the differences in pre-treatment delays (symptom onset to treatment initiation) and proportion with severe illness at diagnosis among PCF- and ACF-detected PwTB in high-risk populations (primary outcomes). We also determined the differences in i) various delays contributing to pre-treatment delay and conditions contributing to severe illness and ii) number of health care provider visits and type of first health care provider visited (secondary outcomes).

## METHODS

This was a cross-sectional analytical study involving primary and secondary data.

### Study setting

India has 768 NTEP districts.^22^ The sub-district level administrative units called TB units represent the lowest level of programme administration. Most public peripheral health institutions that diagnose TB, including primary health centres and above, have a laboratory technician for sputum microscopy and/or nucleic acid amplification tests.^22^ *Ni-kshay* is India’s electronic TB information management system.^23^

In 2023, ACF was conducted in NTEP settings using the algorithm involving symptom screening among high-risk populations, followed by sputum microscopy or rapid molecular tests (Xpert MTB/Rif® or TrueNat®) among symptom-screen positive individuals. Symptom-screen positive was defined as presence of cough or fever of 2 or more weeks duration, unexplained weight loss, chest pain, or haemoptysis. Among clinically vulnerable (see Supplementary Data Table S1), cough or fever of any duration was considered positive.^14,15^ Under guidance from TB units and district TB cells, accredited social health activists, community health workers, or volunteers conducted ACF. One ACF cycle was conducted (intention to screen mapped high-risk population once in the year).^20^ The exact involvement of staff and modes of ACF cycle (campaign mode in a few weeks, divided into multiple rounds, fixed days of a month) varied between states.^19,20^

### Study population, sampling, and sample size

The study population comprised adults (≥15 year) with drug-sensitive pulmonary TB from high-risk populations, notified by the public sector in 2023 from 28 randomly selected NTEP districts across nine states. We selected nine states through stratified random sampling using routinely available composite TB scores measuring NTEP performance and TB burden. Scores above 80 were classified as high performance, 60–80 as medium performance, and below 60 as low performance, with three states randomly selected from each stratum. Of the nine selected states, 30 districts were initially selected using probability proportionate to size sampling based on TB notifications. Two districts were subsequently excluded due to limited administrative support, resulting in 28 study districts (see Supplementary Data Figure S2).

For sampling within study districts, we followed the methodology published earlier as a part of project *Axshya* evaluation during 2016–2017.^11–13^ All people with ACF-detected TB from high-risk populations were enrolled at notification after cross-verification with participants to confirm they were detected through ACF. PCF-detected PwTB from high-risk populations who were notified within (plus or minus) 30 days of an enrolled ACF-detected study participant were eligible for enrolment if ACF activities were not conducted in their residential area 3 months prior to notification. Project research assistants (one in each study district) added the *Ni-kshay* identifiers on an online spread sheet and the central project management unit based in Chennai (southern India) coordinated the verification and study participant enrolment.

Based on expected benchmarks for ACF scale and quality (see Supplementary Data Figure S1),^14,15^ we had planned for ≈1,000 participants in each group. This would provide 80% power to detect a 5-day difference in total pre-treatment delay and a 5% difference in severe illness proportions (alpha = 0.05).^11^ Sample size was restricted by the lower-than-expected yield of ACF (total ACF-detected TB in an ACF cycle divided by expected undetected TB in the high-risk population) in routine programme settings as the scale and quality were below the benchmarks in 2023.^20^ While 18% (benchmark of ≥11%) of population was mapped as high-risk (see Supplementary Data Table S1), the scale of ACF was below the benchmark (7% mapped population screened versus benchmark ≥90%). The number needed to screen was high (4,971 against the conservative benchmark ≤1,538) to detect one person with TB.^20^

### Data collection, variables, and analysis

**FIGURE 1.**
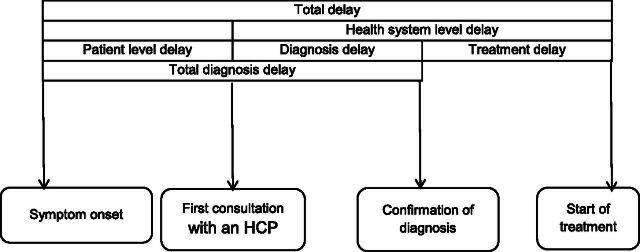
Conceptual framework on definitions of delay before treatment initiation among adults with pulmonary TB. HCP: health care provider. Reprinted with modification from Sreeramareddy CT et al.^32^ with permission from International Union Against Tuberculosis and Lung Disease (The Union), Copyright The Union 2014.

Project research assistants completed in-person interviews and triaging assessments at the earliest opportunity after participant enrolment. Pre-treatment delay data were collected using standardised instruments that included dates of symptom onset and first consultation with health care providers collected as primary data, while diagnosis and treatment initiation dates were extracted from *Ni-kshay* as secondary data (Figure 1**)**. Triaging for severe illness was conducted using a tool recommended in literature and used in Tamil Nadu’s *Kasanoi Erappila Thittam* (meaning TB death-free project in Tamil) differentiated TB care initiative (see Supplementary Data Table S2).^24–26^ PwTB presenting with very severe undernutrition, respiratory insufficiency, or poor performance status were classified as severely ill.^24,27–31^

Single-entered (with data entry checks) primary data in EpiCollect5 mobile application (© 2024 Center for Genomic Pathogen Surveillance, v5.1.52) were merged with the *Ni-kshay* data and analysed using EpiData analysis software (v2.2.2.183, EpiData Association, Odense, Denmark). Statistical analysis included calculation of frequencies with proportions, means with standard deviations, and medians with interquartile ranges. Statistical significance was assessed using χ² tests or *Z* tests for comparing proportions and unpaired *t*-tests or Mann–Whitney *U* tests for comparing continuous variable distributions.

### Ethical statement

The study was approved by the Institutional Human Ethics Committee of ICMR National Institute of Epidemiology (ICMR-NIE), Chennai, India (NIE/IHEC/202201-10 dated 09 February 2022, with renewal on 25 January 2023). Written informed consent was obtained from all study participants and the consent process was approved by the ethics committee.

## RESULTS

### Study participant enrolment

**FIGURE 2.**
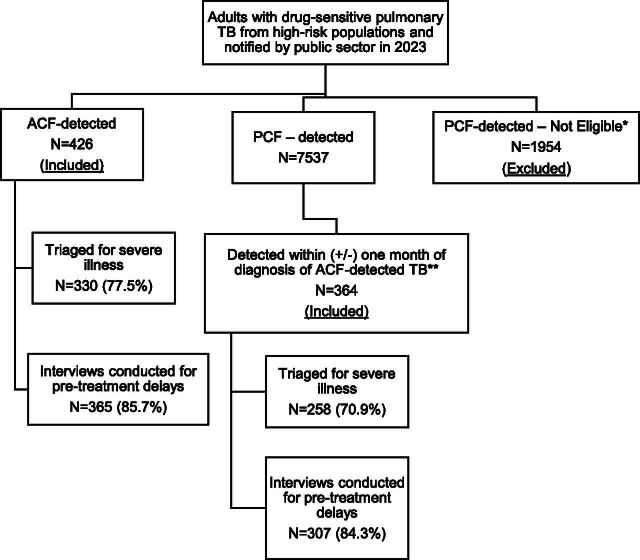
Study participant enrolment and data collection. ACF: active case finding; PCF: passive case finding; *person with TB residing in an area where ACF was conducted in the 3 months prior to diagnosis; **there were instances where there were no PCF-detected PwTB in the district fulfilling the study participant criteria; if there were more than two PCF-detected PwTB per ACF-detected PwTB, we randomly sampled using a 1:1 ratio.

A total of 9,917 adults from high-risk populations were diagnosed with drug-sensitive pulmonary TB and of them 790 study participants were enrolled, 426 ACF-detected and 364 PCF-detected (see Figure 2, Supplementary Data Table S3). Of the 790 enrolled participants, interviews for pre-treatment delays were conducted in 672 (85%) and triaging for severe illness at diagnosis in 588 (74%). The baseline characteristics were not significantly different between those interviewed and those not interviewed for pre-treatment delays (Supplementary Data Table S4). Among those interviewed, the delay (median days, interquartile range) from diagnosis to enrolment and conducting interviews was not significantly different among ACF- and PCF-detected PwTB (24 [8, 64] versus 21 [9, 39], *P* = 0.146). The proportion of ACF-detected TB, diagnosis using rapid molecular tests, and microbiologically confirmed TB was lower among those not triaged when compared to triaged (Supplementary Data Table S4).

### Comparison of baseline characteristics

Among interviewed participants (see Table 1), ACF-detected PwTB showed significantly higher mean age (47.1 years versus 43.9 years), greater median distance from TB diagnosis facilities (8 km versus 6 km), higher proportions of no formal education (52% versus 37%), and lower median annual household income per capita (₹20,000 versus ₹24,000). No significant difference was observed in the use of rapid molecular diagnostic tests (56% among ACF-detected versus 63% among PCF-detected).

**TABLE 1.** Baseline characteristics of public notified adults (≥15 year) with ACF- and PCF-detected drug-sensitive pulmonary TB enrolled from high-risk populations of 28 randomly selected districts in India, 2023.

Variable	Triaged for severe illness at diagnosis (*N* = 588)^A^		*P* value^B^	Interviewed for pre-treatment delay (*N* = 672)^A^		*P* value^B^
	ACF-detected (*N* = 330)	PCF-detected (*N* = 258)		ACF-detected (*N* = 365)	PCF-detected (*N* = 307)	
Age (mean [SD])	47.9 (17.1)	44.2 (16.3)	**0.008**	47.1 (17.1)	43.9 (16.1)	**0.015**
Male sex (*n* [%])	216 (65.5)	185 (71.7)	0.106	244 (66.8)	223 (72.6)	0.105
HIV positive (*n* [%])	1 (0.3)	4 (1.6)	0.092	1 (0.3)	3 (1.0)	0.250
Diabetes mellitus (*n* [%])	26 (7.9)	28 (10.9)	0.212	27 (7.4)	30 (9.8)	0.267
Alcohol use (*n* [%])	107 (32.4)	97 (37.6)	0.154	125 (34.2)	113 (36.8)	0.483
Smoking (*n* [%])	98 (29.7)	79 (30.6)	0.797	103 (28.2)	87 (28.3)	0.977
Diagnosed using rapid molecular test (*n* [%])	157 (47.6)	106 (41.1)	0.116	203 (55.6)	192 (62.5)	0.071
Microbiologically confirmed TB (*n* [%])	260 (78.8)	213 (82.6)	0.253	282 (77.3)	248 (80.8)	0.265
Previously treated TB (*n* [%])	38 (11.5)	35 (13.6)	0.444	43 (11.8)	39 (12.7)	0.723
Residing in urban area (*n* [%])	51 (15.5)	85 (32.9)	**<0.001**	53 (14.5)	91 (29.6)	0.079
Distance (km) from a PHC or above level facility (median [IQR])	5 (2,10)	4 (2,10)	0.076	5 (3,10)	4 (2,10)	0.079
Distance (km) from a TB diagnosis facility (median [IQR])	7 (3,14)	5 (2,10)	**<0.001**	8 (3,15)	6 (3,13)	**0.014**
Education – cannot read or write (*n* [%])	173 (53.2)	88 (35.2)	**<0.001**	190 (52.1)	113 (36.8)	**<0.001**
Education of head of family – cannot read or write (*n* [%])	171 (52.6)	100 (40.0)	**0.002**	186 (51.0)	120 (39.1)	**0.002**
Occupation – unemployed (*n* [%])	24 (7.3)	11 (4.3)	0.128	27 (7.4)	13 (4.2)	0.081
Occupation of head of family – unemployed (*n* [%])	24 (7.3)	12 (4.7)	0.194	27 (7.4)	15 (4.9)	0.183
History of TB (ever) in the family (*n* [%])	78 (23.6)	52 (20.2)	0.290	88 (24.1)	70 (22.8)	0.692
History of TB death (ever) in the family (*n* [%])	21 (6.4)	10 (3.9)	0.148	24 (6.6)	16 (5.2)	0.445
Annual household income (₹) per capita (median [IQR])	21,709 (12,000, 36,000)	25,714 (15,000, 48,000)	**0.004**	20,000 (12,000, 35,571)	24,000 (12,000, 44,500)	**0.046**

Bold values indicate statistically significant differences (*P* < 0.05).

ACF = active case finding; PCF = passive case finding; PHC = primary health centre; IQR = interquartile range.

A Of 790 enrolled, 672 (85%) were interviewed for pre-treatment delays and 588 (74%) were triaged at diagnosis (within 7 days).

B χ² test/*Z* test for comparing proportions, unpaired *t* test or Mann–Whitney *U* test, as applicable.

### Comparison of health care–seeking patterns

Between symptom onset and diagnosis, ACF-detected PwTB had significantly fewer health care provider visits compared to PCF-detected (median one versus two, *P* = 0.011) (Supplementary Data Table S5). In the ACF-detected group, eventual TB diagnosis occurred due to advice from accredited social health activists in 84% of participants. In contrast, among PCF-detected, eventual TB diagnosis was based on advice from government doctors in 30%, accredited social health activists in 18%, and private doctors in 14% of participants (Supplementary Data Table S6). Among ACF-detected, 24% had already visited health care providers between symptom onset and date of ACF activity (data not shown).

### Comparison of pre-treatment delays and severe illness

Both ACF-detected and PCF-detected PwTB had a median pre-treatment delay of 46 days (Table 2). There were no statistically significant differences across various delay components, except for health system–level treatment initiation delay, which was significantly higher in the ACF-detected PwTB (median 2 days) compared to the PCF-detected (median 1 day). The proportion with severe illness among the ACF-detected PwTB was 39%, while it was 34% in the PCF-detected (*P* = 0.180) (Table 3). The distribution of various indicators contributing to severe illness showed no statistically significant differences between groups (Table 3).

**TABLE 2.** Pre-treatment delay among public notified adults (≥15 year) with ACF- and PCF-detected drug-sensitive pulmonary TB enrolled from high-risk populations of 28 randomly selected districts in India, 2023 (N = 672).^A^

Type of delay (in days)	ACF-detected		PCF-detected		P value^C^
	Total assessed	Delay	Total assessed	Delay	
	n^B^	Median (IQR)	n^B^	Median (IQR)	
Patient level (a)	310	30 (10, 70)	292	30 (8, 67)	0.333
Health system level – diagnosis (b)	335	2 (0, 9)	309	1 (0, 21)	0.165
Health system level – treatment initiation (c)	422	2 (1, 5)	360	1 (0, 3)	**<0.001**
Diagnosis delay (a + b)	340	42 (20, 92)	292	43 (21, 92)	0.811
Health system level (b + c)	333	6 (3, 14)	308	4 (2, 21)	0.104
Total pre-treatment delay (a + b + c)	338	46 (24, 95)	291	46 (22, 94)	0.595

Bold values indicate statistically significant differences (*P* < 0.05).

ACF = active case finding; PCF = passive case finding; IQR = interquartile range.

A Of 790 enrolled, 672 (85.1%) were interviewed for pre-treatment delays.

B Both dates available to calculate the corresponding delay among 672 interviewed.

C Mann–Whitney *U* test.

**TABLE 3.** Proportion of severe illness at diagnosis among public notified adults (≥15 year) with ACF- and PCF-detected drug-sensitive pulmonary TB enrolled from high-risk populations of 28 randomly selected districts in India, 2023 (N = 588).^A^

Criteria	ACF-detected (N = 330)	PCF-detected (N = 258)	*P* value
	n (%)^B^	n (%)^B^	
Triage-positive/severely ill (see Supplementary Data Annex S1)	129 (39.1)	87 (33.7)	0.180
Very severe undernutrition related indicator (any one)	48 (14.5)	38 (14.7)	0.950
Respiratory insufficiency related indicator (any one)	100 (30.3)	68 (26.4)	0.293
Inability to stand without support (poor performance status)	15 (4.5)	15 (5.8)	0.488
BMI ≤ 14	43 (13.0)	33 (12.8)	0.944
BMI 14–16 with leg swelling	5 (1.5)	5 (1.9)	0.704
RR > 24/min	72 (21.8)	58 (22.5)	0.848
Oxygen saturation < 94%	47 (14.2)	28 (10.9)	0.221

ACF = active case finding; PCF = passive case finding; BMI = body mass index (kg/m^2^), RR = respiratory rate.

A Of 790 enrolled, 588 (74%) were triaged. ^B^χ^2^ test/Z test for comparing proportions, as applicable.

## DISCUSSION

This is the first nationally representative assessment of benefits of ACF conducted by a national TB programme in terms of early TB detection. In high-risk populations in India, relative to PCF, ACF-detected PwTB were older, resided farther from TB diagnosis facilities, belonged to households with lower education levels and income, and experienced fewer health care provider visits before diagnosis. However, this did not translate into early detection. Among high-risk populations, the burden of severe illness at diagnosis was high.

A strength of our study is that we collected comprehensive data on baseline characteristics. This included use of rapid molecular diagnostic tests that were not significantly different, ensuring that any evidence of early detection (if any) could be attributed to ACF rather than differential diagnostic approaches. The study was conducted in routine operational settings, reflecting real-world ground-level realities of ACF implementation. Several limitations should also be acknowledged. We could not achieve the planned sample size of 1,000 ACF-detected participants due to prevailing suboptimal scale and quality of ACF.^19,20^ Although 15% of enrolled participants were not interviewed for pre-treatment delays, those interviewed and not interviewed had similar baseline characteristics, increasing confidence in internal validity. Among those interviewed, though recall limitation cannot be ruled out, there was no differential recall limitation (no recall bias) between both the groups. A higher proportion of enrolled participants were not assessed for severe illness, and those triaged differed from non-triaged participants on several baseline characteristics, requiring cautious interpretation.

Despite these limitations, three key findings have important implications for policy and practice. First, ACF conducted by NTEP in routine settings was not associated with early detection. This could be attributed to ACF scale and quality indicators being below established benchmarks.^19,20^ In this context of low yield, we speculate that less severely ill undetected PwTB, who typically have shorter symptom duration, may be less likely to get detected and if detected, less likely to complete the ACF care cascade. In future large-scale ACF evaluation, samples size calculations could account for comparison of ACF delays in areas with high and low yield. Second, the national estimates from our study were remarkably similar to estimates from project *Axshya* during 2016–17.^13^ ACF was associated with fewer health care provider visits and detection of more vulnerable PwTB compared to PCF, but did not reduce total pre-treatment delay.^13^ However, unlike project *Axshya*, which demonstrated significant reduction in health system–level diagnosis delay, our study found no such reduction. Between 2016–17 and 2023, some changes are worth noting in high-risk populations. The proportion of ACF-detected participants who had already visited health care providers before ACF activity decreased from 44% to 24%.^13^ Total pre-treatment delay in PCF-detected adults with TB reduced: 57 days in 2016–17 (from eligibility for sputum examination (not symptom onset) to treatment initiation) to 46 days in 2023 (from symptom onset to treatment initiation).^13^ A systematic review published in 2014 reported a pre-treatment delay (assumed to be in general population of India) of 55 days.^32^ Interestingly, in general population 10–20 years ago while 48% people with pulmonary TB first visited a private health care provider, in high-risk populations the same was 57% in 2016–17 and it reduced to 29% in 2023. At the same time, the preference for a public doctor (first visited a public doctor) among adults with TB in high-risk populations increased from 42% to 62%.^13^ Third, limitations notwithstanding, the high burden of severe illness at TB diagnosis among high-risk populations, exceeding 30% in both groups, represents a significant concern. In Tamil Nadu, 12% of all adults with TB (general population) are severely ill at diagnosis.^24^ This provides additional evidence for ACF with high scale and quality, combined with greater utilisation of highly sensitive rapid molecular diagnostic tests in high-risk populations and simple/scalable differentiated care pathways (early referral for severe cases, inpatient/timely care) in line with ongoing Tamil Nadu's *Kasanoi Erappila Thittam* (Supplementary Data Table S2).^24–26^

## CONCLUSION

In the context of ACF being implemented in high-risk populations across all districts under India’s NTEP, though ACF was associated with reduced health care provider visits and identifying the most vulnerable PwTB in 2023, it was not associated with early TB detection. Given that ACF yield remained below established benchmarks, addressing this along with greater use of rapid molecular diagnostic tests in high-risk populations will contribute towards achieving early detection. Based on the six tips for TB ACF published from our qualitative systematic enquiry and other suggested recommendations,^19–21^ the existing TB ACF guidance may be modified.^14,15^ The high burden of severe illness at TB diagnosis in high-risk populations requires immediate attention. India’s Central TB Division is aware of these findings, and steps are being taken to address these programmatic challenges.
